# Supplementary material for: A Flexible Regression Modeling Approach Applied to Observational Laboratory Virological Data Suggests That SARS-CoV-2 Load in Upper Respiratory Tract Samples Changes with COVID-19 Epidemiology
Source: Viruses. 2023 Sep 23;15(10):1988. doi: 10.3390/v15101988 (PMC10610845; doi:10.3390/v15101988)
Supplement: Supplementary file 1 [file viruses-15-01988-s001.zip › final-viruses-2515788 suppl.pdf]

viruses

MDPI

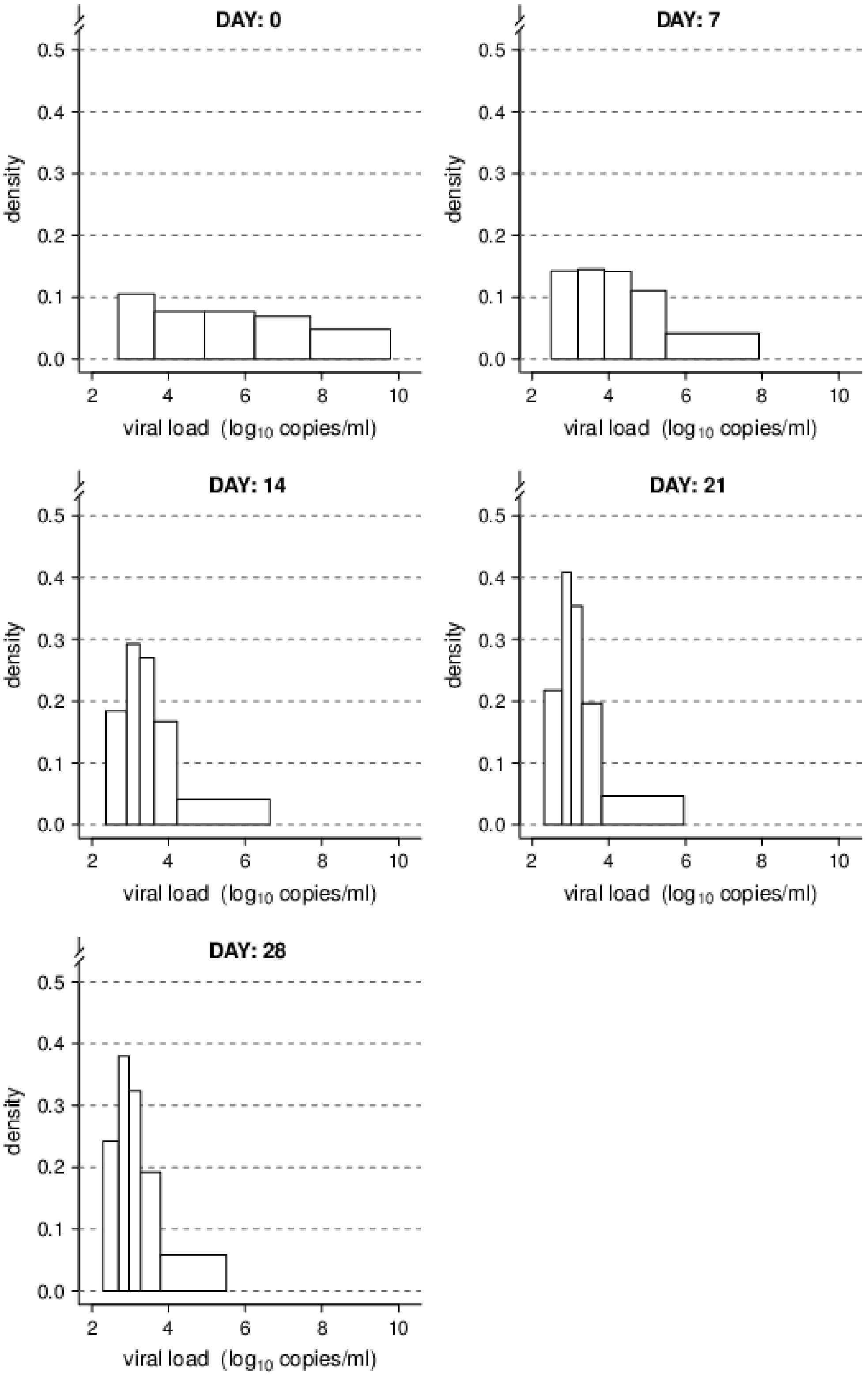

(**A**)

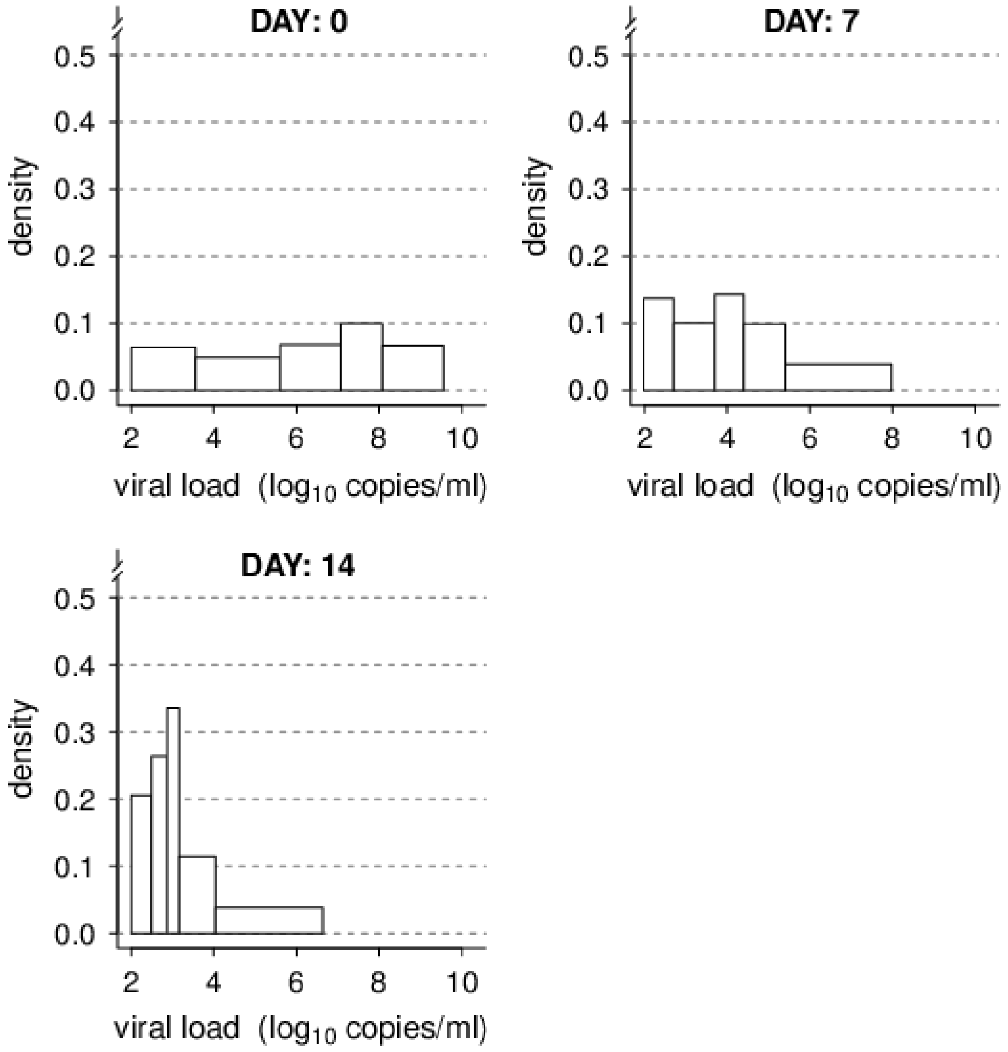

(**B**)

Figure S1. (**A**) Estimated distribution of viral load at fixed days from the episode index for the 2020 winter wave. (**B**) Estimated distribution of viral load at fixed days from the episode index for the 2021 winter wave.

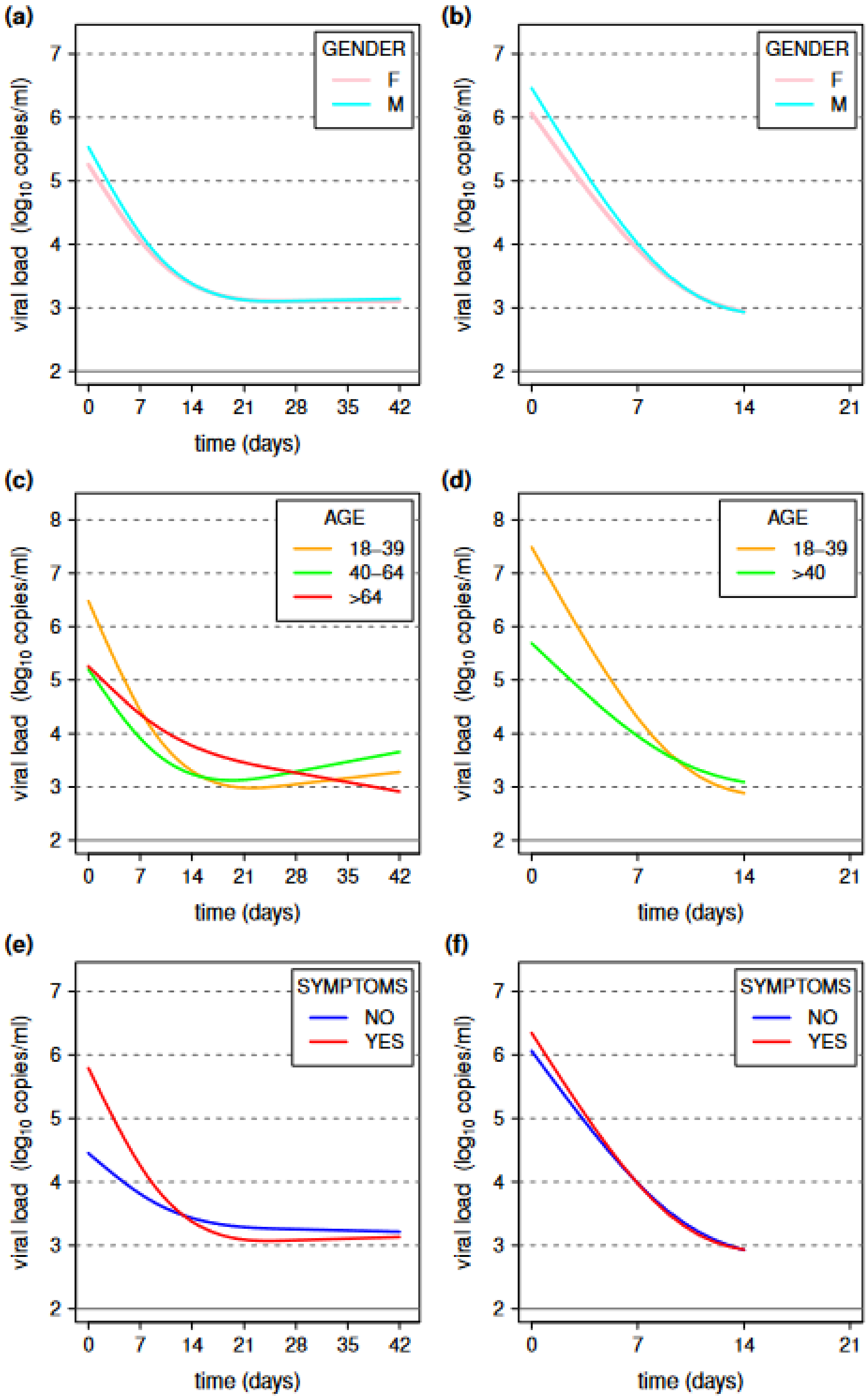

Figure S2. Quantile regression model estimates of the median viral load vs. time (days) of follow up for subgroups: panels (**a**,**c**,**e**) show results for the 2020 winter wave; panels (**b**,**d**,**f**) show results for the 2021 winter wave.
